# Spatial and temporal changes in extracellular elastin and laminin distribution during lung alveolar development

**DOI:** 10.1038/s41598-018-26673-1

**Published:** 2018-05-29

**Authors:** Yongfeng Luo, Nan Li, Hui Chen, G. Esteban Fernandez, David Warburton, Rex Moats, Robert P. Mecham, Daria Krenitsky, Gloria S. Pryhuber, Wei Shi

**Affiliations:** 1 Developmental Biology and Regenerative Medicine Program, Saban Research Institute, Children’s Hospital Los Angeles, Los Angeles, CA 90027 USA; 2 Department of Surgery, Keck School of Medicine, University of Southern California, Los Angeles, CA 90027 USA; 3 Department of Radiology, Keck School of Medicine, University of Southern California, Los Angeles, CA 90027 USA; 4 Department of Cell Biology and Physiology, Washington University School of Medicine, St. Louis, MO 63110 USA; 5 Department of Pediatrics, University of Rochester School of Medicine and Dentistry, Rochester, NY 14642 USA

## Abstract

Lung alveolarization requires precise coordination of cell growth with extracellular matrix (ECM) synthesis and deposition. The role of extracellular matrices in alveogenesis is not fully understood, because prior knowledge is largely extrapolated from two-dimensional structural analysis. Herein, we studied temporospatial changes of two important ECM proteins, laminin and elastin that are tightly associated with alveolar capillary growth and lung elastic recoil respectively, during both mouse and human lung alveolarization. By combining protein immunofluorescence staining with two- and three-dimensional imaging, we found that the laminin network was simplified along with the thinning of septal walls during alveogenesis, and more tightly associated with alveolar endothelial cells in matured lung. In contrast, elastin fibers were initially localized to the saccular openings of nascent alveoli, forming a ring-like structure. Then, throughout alveolar growth, the number of such alveolar mouth ring-like structures increased, while the relative ring size decreased. These rings were interconnected via additional elastin fibers. The apparent patches and dots of elastin at the tips of alveolar septae found in two-dimensional images were cross sections of elastin ring fibers in the three-dimension. Thus, the previous concept that deposition of elastin at alveolar tips drives septal inward growth may potentially be conceptually challenged by our data.

## Introduction

Pulmonary alveoli are organized clusters of sac-like structures distal to the broncho-alveolar duct junction, arising from both the sides and the tips of terminal ducts, which provide the large gas exchange surface that fulfills respiratory function. Growth and maturation of alveoli comprises the final phase of lung development^1,2^, beginning during late gestation (~32 weeks) in human, yet during the early postnatal stage (~5 days) in mice. The alveolarization process is mostly complete around postnatal day (P) 30 in mice, while the end point of human alveogenesis is still somewhat controversial. Alveolar formation is a complex process that requires precise coordination of multiple cell lineages, such as alveolar epithelial type 1 and type 2 cells (AEC1s and AEC2s), together with cell types derived from lung mesenchyme including endothelial cells and myofibroblasts. The precise process of alveolar growth and maturation is not fully known, while prior knowledge is largely based on classic two-dimensional (2-D) structural analysis inferred from histological tissue sections.

Emerging evidence has suggested that the extracellular matrix (ECM) is critically involved in alveolar formation and maturation^3–6^. The ECM is a collection of molecules that are secreted by cells and that surround cells in tissues^7,8^. It is well known that the ECM is a highly organized meshwork that provides mechanical support for tissue integrity and elasticity. Recent evidence also shows that the ECM is in fact an extremely complex scaffold composed of a variety of biologically active molecules, such as growth factors and cytokines, that are sophisticatedly regulated and critical for determining the activities and fates of adjacent cells, including cell adhesion, migration, proliferation, differentiation, and apoptosis. Additionally, ECM molecules may serve as ligands for cell receptors that transmit distinct signals between cells and extracellular environments^9,10^. The ECM is a complex mixture of glycosaminoglycans and proteins, known as the core matrisome. In mammals, approximately 300 proteins constitute the core matrisome, which are usually covalently attached with glycosaminoglycan chains (proteoglycans) or glycans (glycoproteins)^9^. During lung development, these ECM components constantly interact with a variety of lung cells in a coordinated and dynamic manner. The composition and distribution of ECM in lung varies among different developmental stages to ensure proper morphogenesis and maturation. Laminin and elastin are two important ECM components that undergo dramatic changes during alveolar growth.

Laminin comprises a complex of non-collagenous glycoproteins, composed of α, β, and γ chains. Five α, four β, and six γ chains that are encoded by different genes have been identified^11,12^. Laminin, as a key component of basement membrane, is abundant in the developing lungs and plays crucial roles throughout the entire process of lung morphogenesis including alveolar growth by supporting cell growth and interaction, in particular, alveolar capillary network formation and remodeling. Dysregulation of laminin has been reported to cause a decrease in capillary density and impaired distal epithelial/mesenchymal cell differentiation^4,13^, while vascular growth and cell differentiation are essential for alveogenesis.

Elastin fibers meanwhile provide elastic recoil of the lung during breathing. The complex structure of elastin fibers contains two morphologically distinct components: elastin and microfibrils. Fibrillin-based microfibrils are formed pericellularly, with the assistance of several accessory proteins including MFAP, LTBP, versican, ADAMTS, fibronectin, and integrins. Elastin is also assembled pericellularly by clustering of its monomer (tropoelastin), followed by deposition onto a microfibril scaffold and intra-/inter-molecular cross-linking to form insoluble elastin fibers^14,15^. Therefore, elastin fibers are among the most difficult matrix structures to repair because of their size, molecular complexity, and the requirement for numerous helper proteins to facilitate fiber assembly. During alveolar growth, the deposition and dynamic remodeling of elastin fibers at the tip of a secondary septal crest, based on a 2-D image, is assumed to be a driving force for the subdivision of pre-alveolar spaces, leading to the growth of alveolar septa. This hypothesis is supported by the findings that inhibition of elastin protein expression or perturbation of elastin crosslinking causes attenuation or absence of secondary septal crests and thus definitive alveoli^16–18^.

Although three-dimensional (3-D) distributions of important extracellular matrixes such as elastin were examined in adult lung^19^, dynamic changes in ECM structure and components during lung alveolar growth have been studied largely by a 2-D image approach, which has significant limitations in depicting the true 3-D structure of the ECM network^5^, in particular, for ECM (such as laminin and elastin) that undergoes substantial rearrangement and remodeling during alveolar formation. As part of the LungMAP consortium (LungMAP.net) project, we have studied the expression and distribution of laminin and elastin proteins in mouse developing lung by a 3-D imaging approach to better understand ECM structure and its roles in alveogenesis. Additionally, 2-D dynamic analyses of ECM protein distributions in human lungs at different developmental stages were performed.

## Results

### Dynamic changes of laminin distribution and structure in developing lung alveoli

**Figure 1:**
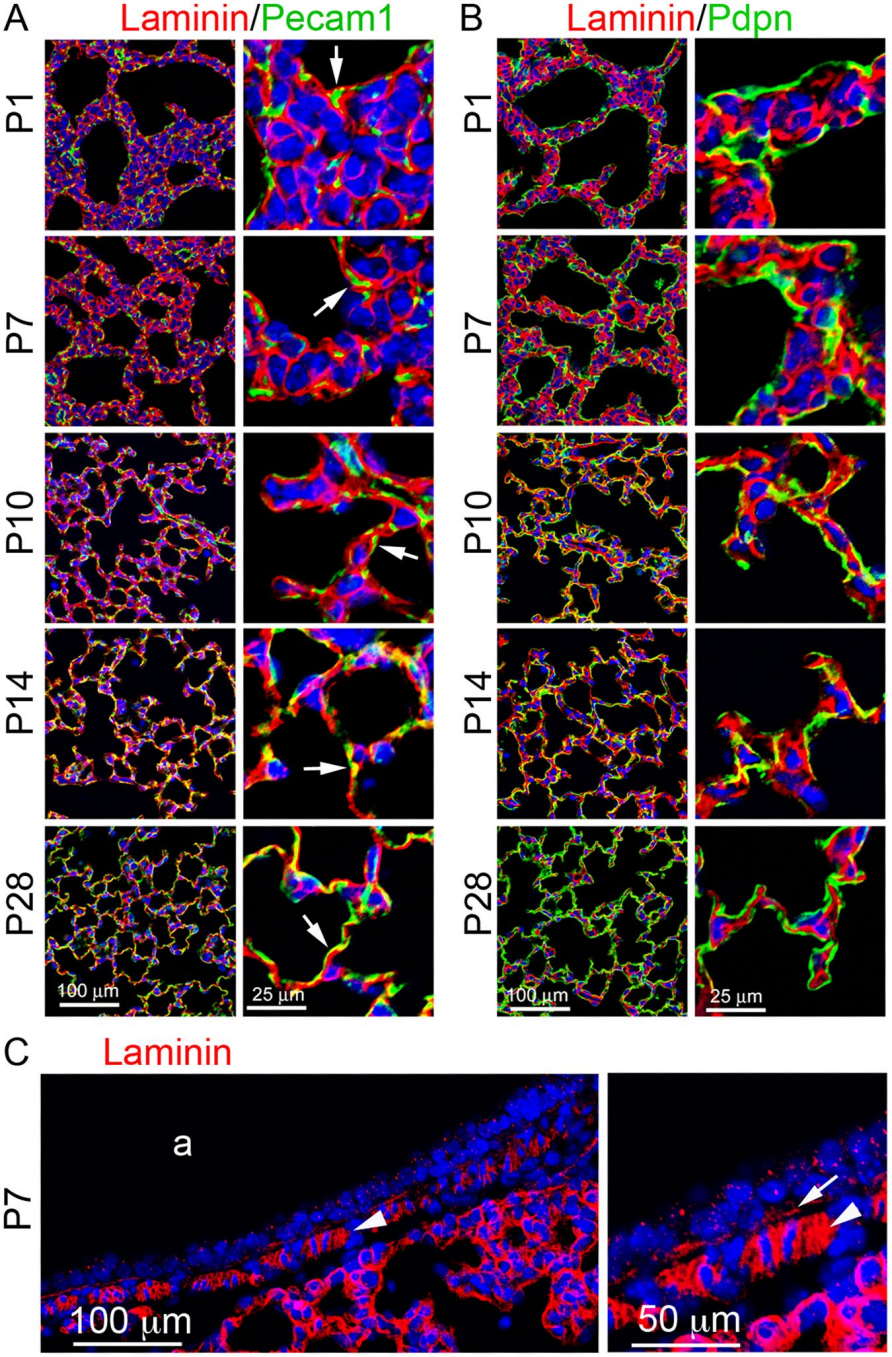
Immunofluorescence staining of lung tissue sections of mice from P1 to P28: (**A**) Laminin (red)/Pecam1 (green), Pecam1 positive cells adjacent to laminin are indicated by arrows. (**B**) Laminin (red)/Pdpn (green). (**C**) Laminin staining of P7 airway (a). Pericellular basement membrane of airway smooth muscle cells and airway epithelial basement membrane are indicated by arrowhead and arrow, respectively.

**Figure 2:**
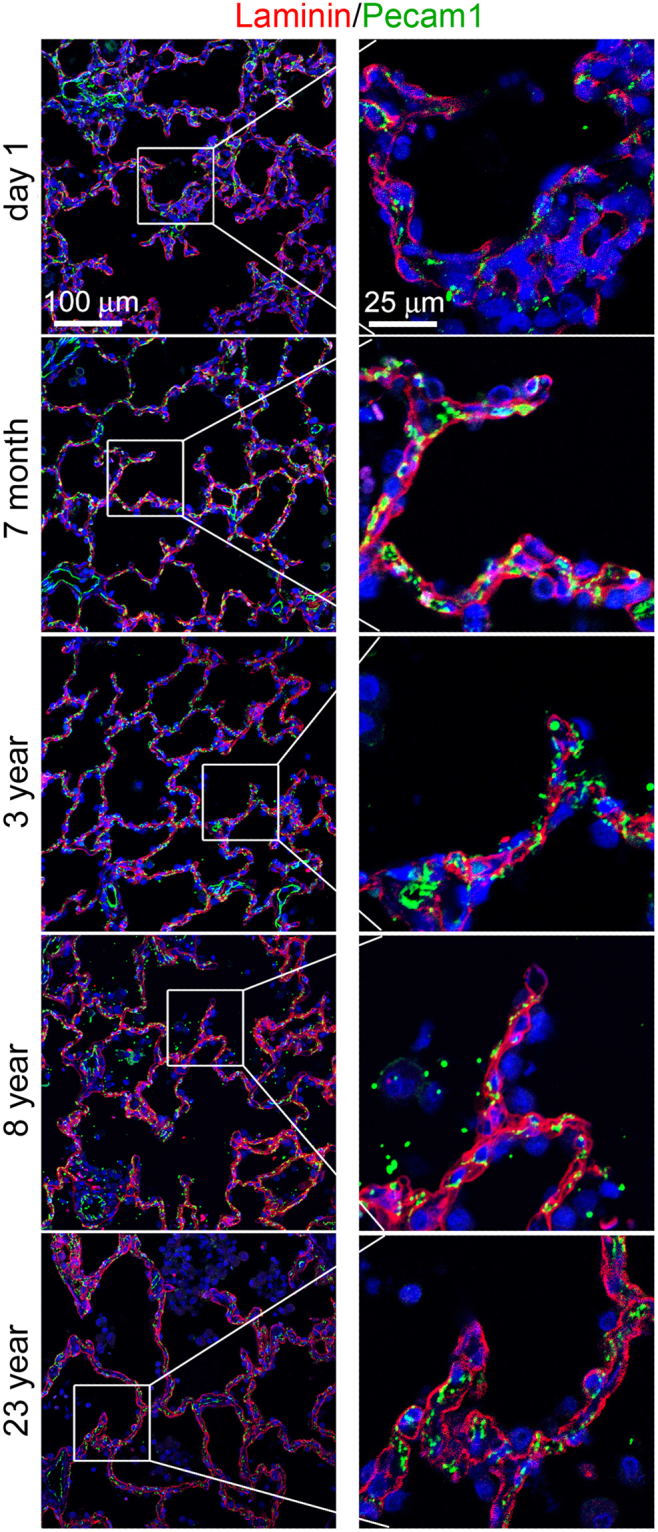
Immunofluorescence staining of laminin (red)/Pecam1 (green) of human lung tissue sections from ages of postnatal day 1, 7 months, 3 years, 8 years, and 23 years. Right panels show magnified boxed areas highlighted in left panel.

Laminin is known to be essential for lung development including alveogenesis. The precise distribution and dynamic changes of laminin protein in the developing lung have not been carefully examined. Laminin protein expression in mouse lungs at different alveolarization stages (P1, P7, P10, P14, and P28) was detected by immunofluorescence staining in thin tissue sections. The relationship between laminin and adjacent cells was compared by co-staining with Pdpn and Pecam1 for type I alveolar epithelial cells (AECI) and endothelial cells, respectively. As shown in Fig. 1, laminin was found extensively within the interstitial space as a cross-linked mesh network, underlying both Pecam1-positive cells (endothelia) and Pecam1-negative cells in primary alveolar septa of P1 lung. Lack of detection of intracellular laminin is likely due to its rapid cellular export after biosynthesis^20^. Laminin was also detected in pericellular spaces of smooth muscle cells underneath the airway epithelium (Fig. 1C), with much stronger staining than in the basement membrane of airway epithelia, suggesting a further role in airway smooth muscle cell growth. During secondary alveolar growth and maturation, the laminin network became progressively simplified (P7 to P14), consistent with the thinning of the alveolar septa. At P14 and P28, laminin appeared to localize more closely to the capillaries than the alveolar epithelium as demonstrated by Pecam1 or Pdpn co-staining (Fig. 1A,B). Similarly, laminin staining in human lung alveoli appeared as intermingled lines from postnatal day 1 to adult (Fig. 2), with many “loop” like structures on the tips of alveoli after 7 months.

### Three-dimensional representation of laminin structure in distal lung of neonatal mice

**Figure 3:**
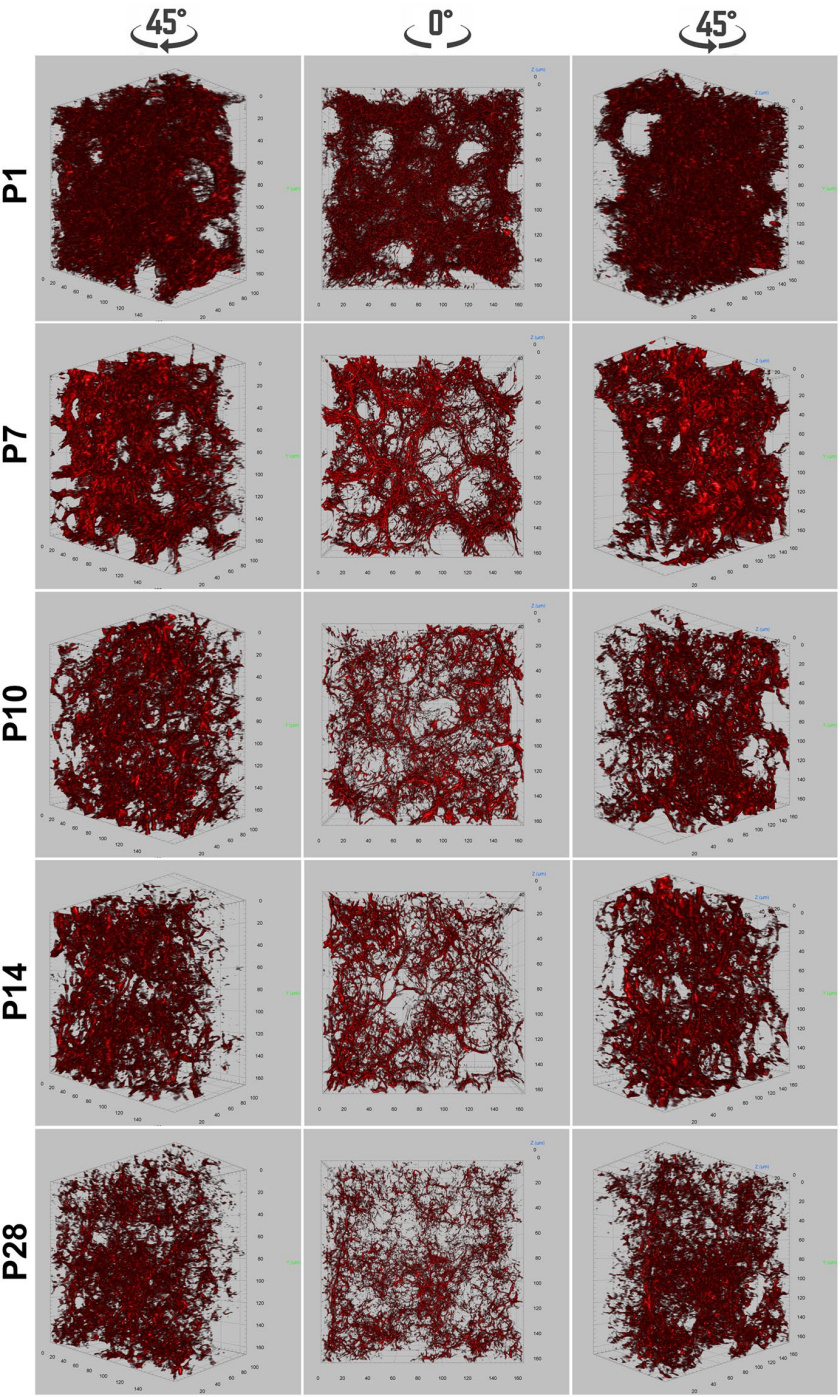
Whole mount immunofluorescence staining of laminin (red) for mouse lungs of P1, P7, P10, P14, and P28. Pictures of 3-D volumetric rendering are presented with views from different angles.

The conventional 2-D images only depict the distribution of laminin and its spatial relationship with adjacent cells in a 2-D cross section, lacking information about its integral 3-D structure. Using whole mount immunofluorescence staining and volumetric image rendering, we characterized the 3-D structure of laminin in mouse lungs undergoing alveogenesis. As shown in Fig. 3 and videos 1–5, the laminin formed a very condensed and complex 3-D network, which we take fancifully as an analogy to resemble the steel rebar and mesh used for concrete reinforcement. Consistent with 2-D observations, the laminin structure within the alveolar walls became progressively simplified during the process of alveolar growth and maturation, which was validated by quantitative measurement of stained laminin volumes (Supplementary Fig. 1A). A continuous network of laminin in the P7 alveolar region was also demonstrated by serial optical planes (Video 6).

### Developmental changes of elastin expression and distribution in distal lung

**Figure 4:**
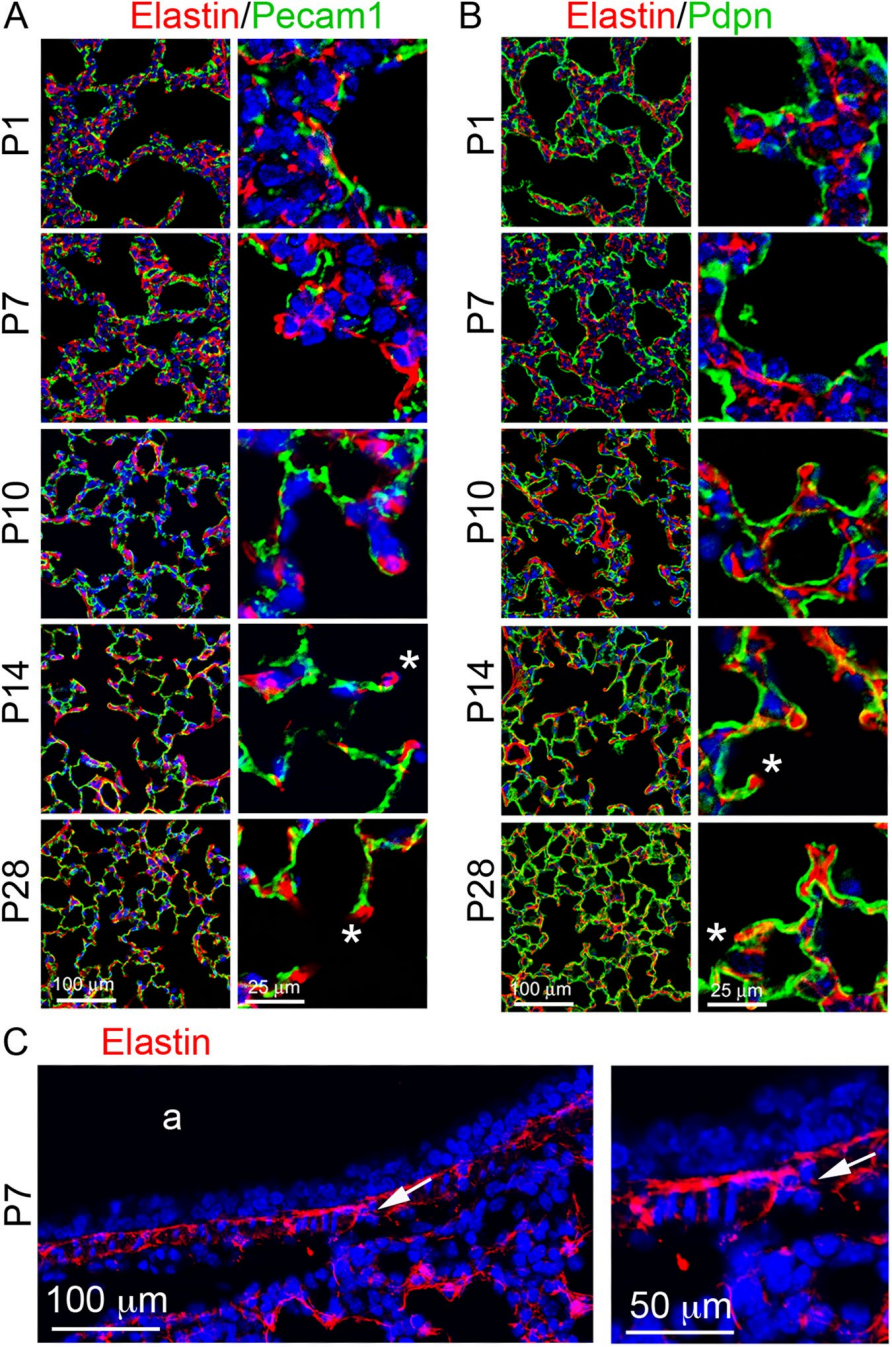
Immunofluorescence staining of lung tissue sections of mice from P1 to P28: (**A**) Elastin (red)/Pecam1 (green). (**B**) Elastin (red)/Pdpn (green). *“finger-like” alveolar protrusions. (**C**) Elastin staining of P7 airway (a). Airway  smooth muscle cell layer is marked by arrows.

Elastin was detected in the alveolar septa of mouse lung tissue sections, appearing in the “fiber, dot and patch” pattern that has been extensively described previously in 2-D in the literatures^21,22^. Unlike laminin, the relative density of elastin staining was increased from P1 to P28 in alveoli, accompanied by increased thickness of elastin fibers (Fig. 4A,B). Towards the end of alveolarization in mice (P28), most elastin fibers were closer to the lining AECI, in contrast with the distribution of laminin that lies closer to endothelial cells. In addition, the elastin matrix was significantly rich at the tip of “finger-like” alveolar protrusions in lungs of all examined stages. It is widely believed that the “finger-like” protrusions are the growing secondary septum that subdivides an established alveolus into two “daughter” alveoli, and the elastin in the alveolar ridges is present as isolated patches or dots at the tip is a driving force of alveolar septation^23^. In addition, elastin fibers were also present in the airway walls, in the mucosa between epithelia and airway smooth muscle cell layer and in the submucosa region (Fig. 4C).

**Figure 5:**
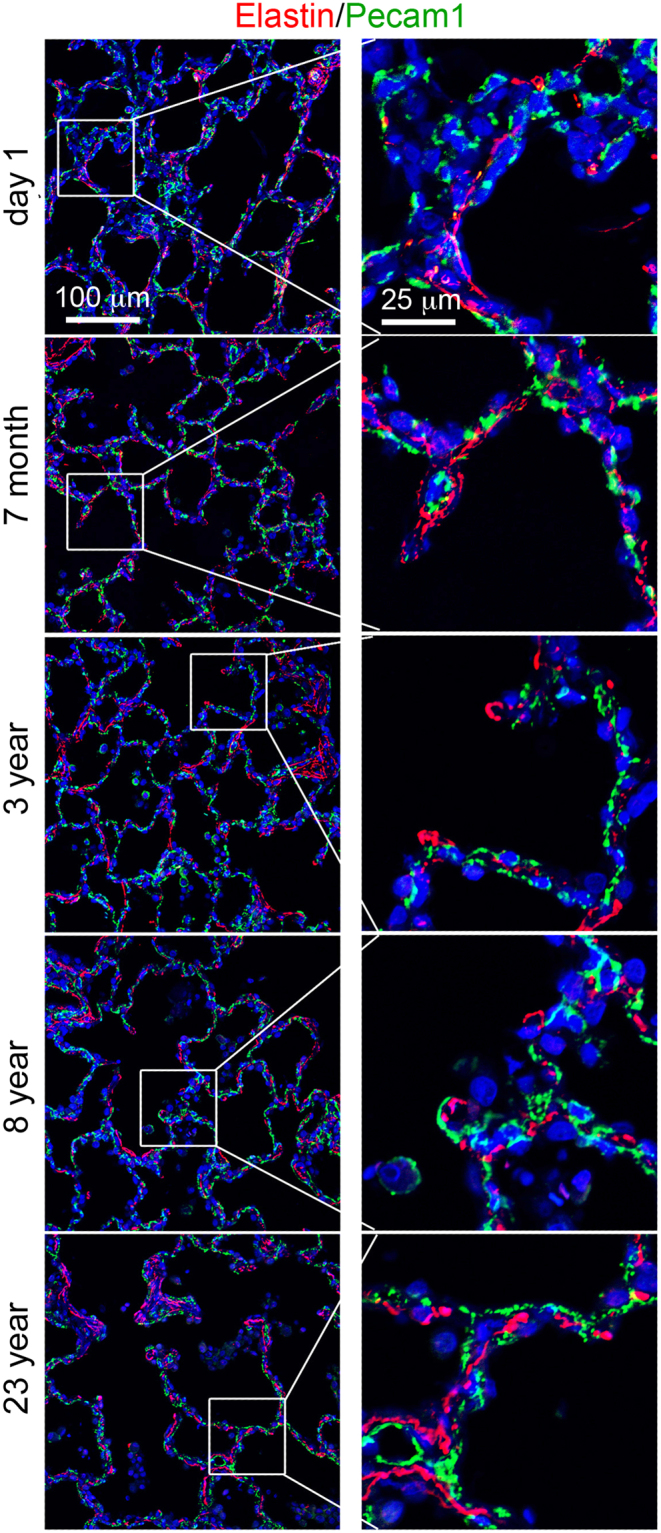
Immunofluorescence staining of elastin (red)/Pecam1 (green) of human lung tissue sections from ages of postnatal day 1, 7 months, 3 years, 8 years, and 23 years. Right panels show magnified boxed areas highlighted in left panel.

Similar to elastin distribution in mouse lung, elastin in developing human lung alveolar septa appeared as “fiber, dot, and patch” (Fig. 5), and was enriched in the tips of protrusions. There was no significant difference in elastin expression patterns from 7 months to young adult (23 years age), although elastin expression was relatively sparse in human lungs at postnatal day 1.

### Visualization of 3-D elastin structure in distal lung of neonatal mice

**Figure 6:**
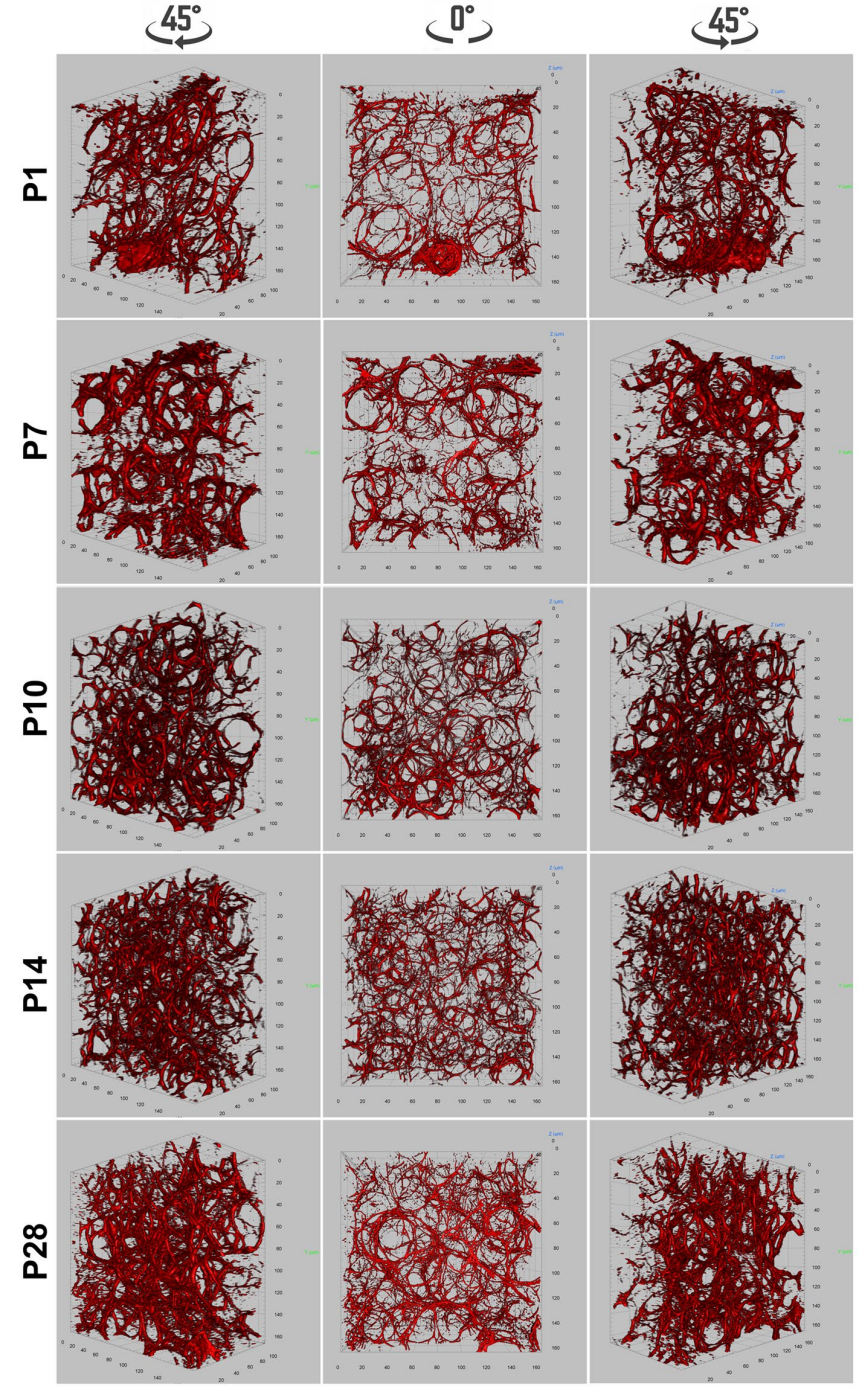
Whole mount immunofluorescence staining of elastin (red) for mouse lungs of P1, P7, P10, P14, and P28. Pictures of 3-D volumetric rendering are presented with views from different angles.

**Figure 7:**
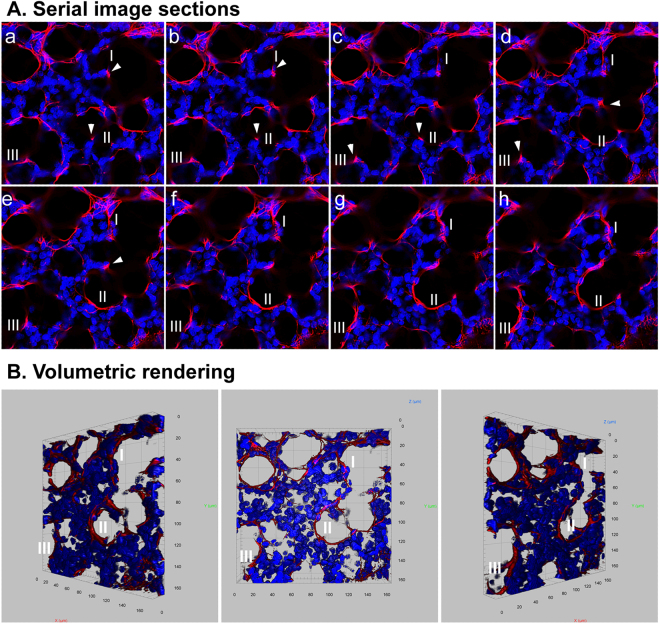
Whole mount immunofluorescence staining of elastin (red) for P7 mouse lung. (**A**) Stack of serial image sections of elastin staining. (**B**) Volumetric rendering of the serial images shown in A. DAPI counterstaining (blue) was used to display the structures of the lung tissue. “I to III” indicated three elastin rims. Arrowheads indicate the patches of elastin present at the tips of alveolar protrusions.

Using whole mount immunofluorescence staining, 3-D elastin structures in developing mouse lung alveoli were analyzed. In P1 neonatal lungs, elastin fibers were mainly localized to the saccular openings of nascent alveoli, forming a ring-like structure (Fig. 6 and Video 7). Throughout subsequent secondary alveolarization and maturation (P7 to P28), the number of such ring-like structures increased, while the relative size of the rings decreased (Fig. 6, Videos 8–11). By quantifying the volumetric signals of elastin staining, the density of elastin in parenchymal tissues showed continuously increased during alveolarization (Supplementary Fig. 1B). The elastin rings were also interconnected between each other via additional elastin fibers (Fig. 7 and Video 12). Isolated elastin patches and dots observed in 2-D images were rarely seen in the 3-D rendering. By analyzing serial optical sections (Fig. 7 and Video 12), those patches and dots of elastin expression were revealed to actually be cross sections through elastin fibers, occurring at different angles. Thus, the description of elastin in alveolar septa as “dot and patch” does not reflect the actual elastin structure, due to limited information from 2-D imaging.

## Discussion

Lung alveolarization is the ultimate phase of lung development that allows growth of gas-exchange surface area to meet gas exchange demands. Alveolar septal walls undergo a process of thinning to minimize the distance that must be traversed by the gases. Complexity in alveogenesis results from coordinated growth of multiple components including epithelial cells, microvascular endothelial cells, interstitial cells, and ECM^3,13^.

ECM is classified into two distinct types based on its location and composition: the interstitial connective tissue matrix and the basement membrane. The basement membrane is a thin layer of ECM between the epithelia/endothelia and surrounding stromal cells, and is composed of two molecular networks: laminin and type IV collagen^24^. These two molecular networks are linked by nidogen and heparan sulfates. The laminin network is anchored to the cell surface by interacting with a variety of ECM molecules, such as integrins, α-dystroglycan, and sulfated glycolipids/sulfatides. The basement membrane provides mechanical support, separates different cell types, as well as signals for cell differentiation, migration, and survival^25^. The functions of basement membranes vary in different organs and at different developmental stages, depending on expression of different isoforms of laminin and Col4 as well as other ECM molecules. The laminin antibody used in this study recognizes multiple isoforms, as it was developed using the laminin isolated from a rat yolk sac tumor cell line^26^. By examining laminin protein expression patterns during lung alveolarization by 2-D and 3-D imaging, the laminin network at the basement membrane in alveoli appears similar to the “steel mesh” inside the concrete of a building, while it becomes more tightly associated with alveolar capillary endothelial cells in mature lung alveolar septa. As mentioned above, there are many isoforms of laminin with dynamic expression changes during lung development^13^. One of the prominent changes of the laminin networks is its reduced complexity that occurs along with the thinning of septal walls. Although it is believed that the alveolar walls become thinner mainly by the apoptosis of interstitial fibroblasts, the remodeling of the laminin in this process should not be overlooked. Further analysis with antibodies targeting specific laminin isoforms will give insight into the specific role of each expressed isoform in lung alveogenesis. For example, laminin γ1 is the most widely expressed laminin chain, and 10 of 15 laminin trimers are composed of laminin γ1. Mice with a mutation in laminin γ1 or defective laminin assembly develop respiratory failure with lungs that have normal differentiation of alveolar type II cells but thickened mesenchyme^27,28^. In addition, stronger laminin staining was found in pericellular spaces of airway smooth muscle cells, which is distinct from the basement membrane structure immediately underneath airway epithelial cells. Consistent with *in vitro* data^29^, this may suggest that laminin plays a key role in airway smooth muscle cell differentiation and/or maintenance.

Elastin fibers and collagen fibers are interwoven with glycosaminoglycans and proteoglycans in the lung interstitial matrix^19^. The essential role of elastin in distal alveolar formation has been evidenced by disrupting elastin synthesis or crosslinking in mouse models. Mice with a homozygous mutation of elastin gene developed fewer and more dilated distal air sacs with attenuated tissue septa prior to their death at postnatal day 4^16^. It is believed that myofibroblasts are the major cell type producing elastin during lung alveolarization^5,30,31^, while platelet-derived growth factor receptor-α (Pdgfra) positive cells are the progenitors of alveolar myofibroblasts. Profound deficiencies in alveolar myofibroblasts and elastin matrix were found in the platelet-derived growth factor (Pdgf) mutant lung, with defects in secondary septa and impaired alveolar formation^17,18^. Furthermore, single cell RNA-seq data (available from Lung Gene Expression Analysis Web Portal (LGEA), https://research.cchmc.org/pbge/lunggens/mainportal.html) also suggest that myofibroblasts are the major cell population expressing *elastin* (*Eln*) during alveolar growth. Perturbation of elastin deposition through inhibiting the crosslinking enzyme lysyl oxidase (LOX) leads to defective alveolar morphogenesis in neonatal mice^18^.

The exact mechanisms by which elastin contributes to alveogenesis remain unknown, so that precise characterization of the location and structure of elastin fibers might provide important answers. As shown in the 2-D images, elastin partially rims alveoli lined by AEC1s, or presents as an isolated dot/patch at the tips of the “finger-like” protrusions that were recognized as the growing septa. While the elastin fibers surrounding the already formed alveoli are believed to provide tissue elasticity that allows the alveoli to stretch during inhalation and spring back during exhalation, the elastin in the tips of the protrusions is thought to be the driving force for nascent septa formation. However, 3-D analysis showed that the isolated patches in the tips of the “finger-like” protrusions were not actually isolated. They were parts of continuous elastin fibers that rim the alveolar opening (Fig. 7 and Video 12). Thus the patch-like elastin pattern in 2-D image is misleading, and is an artifact of oblique sectioning that is absent in 3-D images. By using a 3-D approach, Branchfield *et al*., have also demonstrated that “finger-like” protrusions do not exist during the formation of alveoli^5^. Therefore one of the previously widely accepted concepts that alveogenesis is the process of repeated sub-division of existing alveoli driven by myofibroblasts and elastin deposition is challenged by our 3-D data. Previous studies by us and other groups suggest that intra-saccular pressure and deformation of epithelial cells and/or mesenchymal cells may be one of the main driving forces for distal tip growth and new alveolar formation^32,33^.

The dynamic change of deposition and structural architecture of elastin during alveolar formation was also examined. 3-D rendering showed that the elastin rings were becoming smaller, but more condensed as alveogenesis proceeds. These changes of elastin structure closely correlate with the reducing size and increasing number of alveoli. A number of studies have demonstrated that the elastin network is present prior to alveolar formation, and is then remodeled to form thick elastin bundles specifically encircling alveolar openings during the rapid phase of alveogenesis, when abundant myofibroblasts are colocalized to the ridges of alveoli^5,23^. After P14, alveolar myofibroblasts rapidly decline while the elastin network persists. This suggests that alveolar myofibroblasts are not the only cell type contributing to elastin production in alveoli, however, they may play a critical role in remodeling elastin fibers into a structural framework that supports alveolar growth.

## Materials and Methods

### Mouse strains, breeding and collection of lung specimens

All mice were bred in a C57BL/6 strain background. Sexually mature mice (6~8 weeks) were mated, and the age for newly delivered pups was designated as postnatal day (P) 1. Lungs were then collected from mice at P1, P7, P10, P14, and P28. For whole mount immunofluorescence staining and 3-D imaging, lungs from P1 mouse were isolated and fixed in DMSO:methanol (1:4) overnight at 4 °C, while lungs from P7 to P28 mice were perfused and flushed by injecting PBS through the right ventricle, followed by intra-tracheal instillation of DMSO:methanol (1:4) under 25 cm H_2_O pressure. Lungs were then isolated and submerged in DMSO:methanol (1:4) overnight at 4 °C. For preparation of regular paraffin tissue sections and 2-D histological examination, isolated lungs were dissected and fixed in 4% paraformaldehyde, as described in our previous publication^34^. All mouse experimental procedures were conducted in accordance with NIH Guide, and approved by the Institutional Animal Care and Use Committee of Children’s Hospital Los Angeles.

### Human lung tissue samples

Normal lung tissue paraffin sections of postnatal day 1, 7 months, 3 years, 8 years, and 23 years were obtained from the Biorepository for Investigation of Neonatal Diseases of the Lung at the University of Rochester Medical Center, the LungMAP human tissue core. The quality lung tissue was processed by inflation fixation, to 25 cm H2O with 10% buffered formalin, of the right lower lobe of donor organs provided via the National Disease Research Interchange (NDRI) and the International Institute for Advancement of Medicine (IIAM) through the United Network of Organ Sharing, the non-profit organization that manages the national Organ Procurement and Transplantation Network (OPTN). Family informed consents were obtained for research for all donated organs. The work was conducted in accordance with NIH Guide, and approved by the University of Rochester Medical Center Institutional Review Board.

### Histology and immunofluorescence analysis on regular 2D slides

Paraffin sections (4 μm-thick) were used for 2-D immunofluorescence staining following our published methods^34,35^. The rabbit anti-mouse elastin antibody and rabbit anti-human elastin antibody were generated by Dr. Robert Mecham at Washington University, St. Louis, and rabbit anti-laminin antibody was purchased from Thermo Scientific (#RB-082). The specificities of these antibodies were validated as reported in a previous publication for anti-elastin antibodies^36^ as well as shown in Supplementary Fig. 2 for anti-laminin antibody. Specific cell types were identified by co-immunofluorescence staining of related molecular markers, including Pdpn (Clone 8.1.1, The Developmental Studies Hybridoma Bank) and Pecam1 (#sc-1506, Santa Cruz Biotechnology). Images were captured using a Zeiss LSM 710 confocal microscope at the Cellular Imaging Core Facility of the Saban Research Institute.

### Whole mount immunofluorescence staining, 3-D imaging and image processing

The fixed lungs were gradually rehydrated and embedded in a gelatin/albumin solution mixed with formaldehyde. After becoming solid, the gel-like blocks were then subjected to sectioning with a thickness of 300 μm using vibratome^37^.

Whole mount immunostaining was performed using a modified protocol by increasing incubation temperature and prolonging incubation time to improve antibody penetrance in large tissues^38–40^. Briefly, the lung specimens were blocked with 5% donkey serum in PBS/2% Triton-100 (PBST) overnight at 37 °C, with gentle rocking. The lung specimens were then incubated with primary antibody diluted 1:1000 in blocking solution at 37 °C, with gentle rocking for one week. Antibodies used in the whole mount staining included anti-laminin and anti-elastin antibodies were described above. After washing in PBST at room temperature for five times, the lung specimens were incubated with the secondary antibodies (diluted 1:1000 in PBST)/DAPI (0.5 μg/ml) at 37 °C, with gentle rocking for three days. Following extensive washing in PBS, lung specimens were dehydrated by gradually replacing PBS with methanol and then cleared using a 1:2 (v/v) mixture of benzyl alcohol/benzyl benzoate (BABB) prior to imaging.

Serial optical sections were acquired using a confocal microscope (Zeiss LSM 710) equipped with a Plan-Apochromat 20x/0.8 M27 objective lens with 2x electronic zoom to reach a resolution of 0.208 μm per pixel. The interval between successive planes was set to 1 μm. Raw data were deconvolved to correct optical distortions using AutoQuant X software with the adaptive point spread function (blind) method and default parameters. 3-D rendering (volumetric rendering), visualization, segmentation and morphological analysis were performed using Arivis Vision4D software (Version.2.12.3, Arivis AG, Munich, Germany), a 3D/4D image processing software program^41^. For quantitative analysis of a cubic imaging block generated by 3-D rendering, the volume occupied by the fluorescent signal of different staining (DAPI, laminin, or elastin) was thus computed as the size of segmented voxels of the segmentation from the cubic blocks^42^. The quantitative data were expressed as mean ± SD and analyzed using one-way ANOVA and Pairwise Comparison by VassarStats program. P < 0.05 was considered statistically significant.

All data generated during this study are included in this published article and its Supplementary Information files.

## Electronic supplementary material

Supplementary Figures

Video 1

Video 2

Video 3

Video 4

Video 5

Video 6

Video 7

Video 8

Video 9

Video 10

Video 11

Video 12
