# Supplementary material for: Spatial and temporal changes in extracellular elastin and laminin distribution during lung alveolar development
Source: Sci Rep. 2018 May 29;8:8334. doi: 10.1038/s41598-018-26673-1 (PMC5974327; doi:10.1038/s41598-018-26673-1)
Supplement: Supplementary file 1 — Supplementary Figures [file 41598_2018_26673_MOESM1_ESM.pdf]

Spatial and temporal changes in extracellular elastin and laminin distribution during lung alveolar development

Yongfeng Luo, Nan Li, Hui Chen, G. Esteban Fernandez, David Warburton, Rex Moats, Robert P Mecham, Daria Krenitsky, Gloria S Pryhuber, Wei Shi

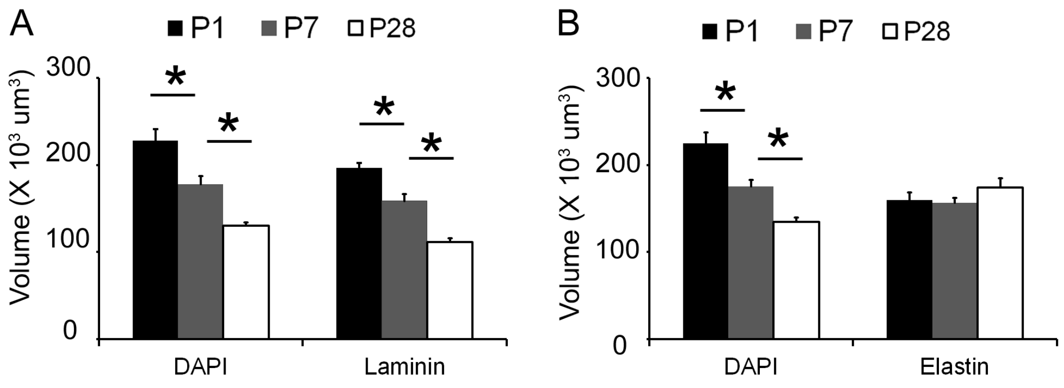

Supplementary Figure 1. Quantitative changes of laminin and elastin at different alveolarization stages were analyzed by volumetric measurement of fluorescence signals from anti-laminin immunostaining (A), or anti-elastin immunostaining (B), within a cube ($2{,}769\text{x}10^3$ μm^3) reconstructed by 3-D rendering. The parenchymal tissue volume in the same cube was estimated by measuring the volume of DAPI-counterstained cell nuclei. *$P<0.05$. The relative density of laminin (the ratio of laminin to DAPI) remained unchanged, while the relative density of elastin (the ratio of elastin to DAPI) was increased from P1 to P28.

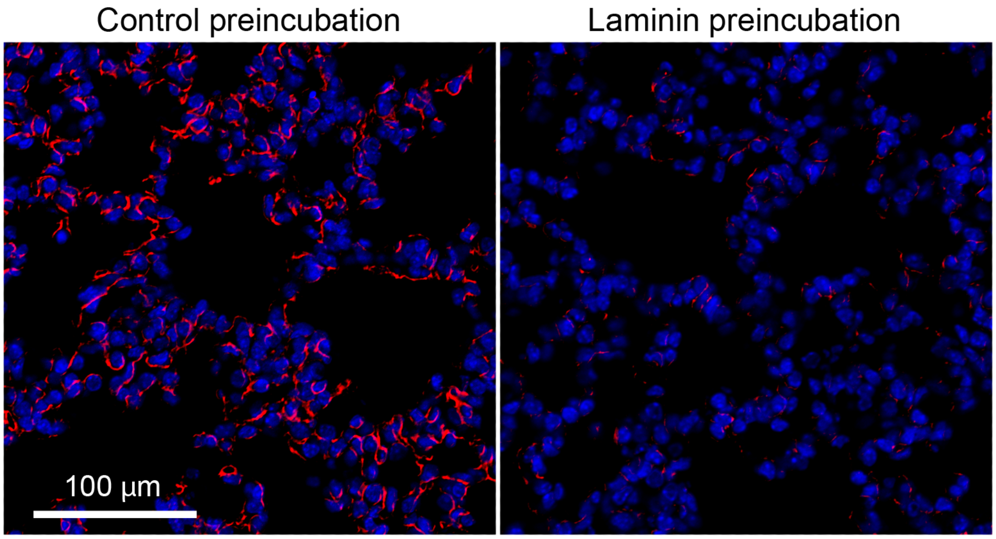

Supplementary Figure 2. Validation of anti-laminin antibody specificity. The laminin antibody diluted 1:200 in working solution was pre-incubated in a laminin coated plastic dish or an uncoated plastic dish (control) overnight at 4°C. The antibodies were then used for P7 lung tissue immunofluorescence staining, shown as red signal. Cell nuclei were counterstained with DAPI (blue).
